# Predicting Chemotherapy Benefit across Different Races in Early-Stage Breast Cancer Patients Using the Oncotype DX Score

**DOI:** 10.3390/cancers15123217

**Published:** 2023-06-16

**Authors:** Vikram R. Shaw, Christopher I. Amos, Chao Cheng

**Affiliations:** 1 Institute for Clinical and Translational Research, Baylor College of Medicine, Houston, TX 77030, USA; vikram.shaw@bcm.edu; 2 Section of Epidemiology and Population Sciences, Department of Medicine, Baylor College of Medicine, Houston, TX 77030, USA; 3 Dan L Duncan Comprehensive Cancer Center, Baylor College of Medicine, Houston, TX 77030, USA

**Keywords:** oncotype DX, breast cancer, prognosis, chemotherapy sensitivity

## Abstract

**Simple Summary:**

The Oncotype DX recurrence score is widely used to help clinicians treat patients with early-stage breast cancer. However, the threshold at which the Oncotype DX score is used to guide chemotherapy treatment may vary for different patient groups, and the present study describes a machine learning method to quantitatively determine the optimal chemotherapy sensitivity threshold. Utilizing publicly available breast cancer survival data, we demonstrated that 2.05–2.72x more lymph-node-negative and 2.08–5.02x more lymph-node-positive patients may benefit from receiving chemotherapy at a lower Oncotype DX score than current guidelines (RS > 25 or RS > 30) suggest. Additionally, our models indicate a racial difference in chemotherapy benefit that may help physicians provide tailored treatment to specific patients. Specifically, white, black, and Asian women with early-stage ER+/HER2−/LN− tumors may benefit from chemotherapy when their Oncotype DX scores are greater than 19.9, 37.2, and 18.0, respectively.

**Abstract:**

Background: Oncotype DX assay, a multigene molecular test, has been widely used to stratify relapse risk and guide chemotherapy treatment in breast cancer. However, the optimal threshold of the Oncotype DX score in predicting chemotherapy benefit and its racial variation has not been investigated. Methods: In this study, we apply a random forest survival model to the SEER-Oncotype cohort data (Surveillance, Epidemiology, and End Results with Oncotype DX test information for breast cancer patients) and determine chemotherapy benefit thresholds in early-stage, estrogen-receptor-positive (ER+), and HER2-negative (HER2−) patients of different races. Results: Our results indicate that early-stage ER+, HER2−, and LN−/LN+ patients may benefit from receiving chemotherapy at a lower Oncotype DX score than current guidelines (Recurrence Score, RS > 25 or RS > 30) suggest. According to the estimated chemotherapy sensitivity thresholds from our models, 2.05–2.72-fold more lymph-node-negative (LN−) and 2.08–5.02-fold more lymph-node-positive (LN+) patients who may not currently be recommended for chemotherapy by their Oncotype DX test result may actually have the potential to benefit from chemotherapy. Furthermore, our models indicate a racial difference in chemotherapy benefit: white, black, and Asian women with early-stage ER+/LN− tumors benefit from chemotherapy when their Oncotype DX scores are greater than 19.9, 37.2, and 18.0, respectively. Conclusions: Our study provides a method for calibrating multigene molecular tests to help guide treatment decisions in racially and ethnically diverse patients with cancer. Specifically, we identify key chemotherapy sensitivity thresholds for the Oncotype DX recurrence score test in breast cancer patients and provide evidence that certain patients may benefit from receiving chemotherapy at a lower threshold than the current clinical guidelines suggest.

## 1. Introduction

Breast cancer is the most common malignant tumor in women worldwide [1,2]. The adoption of biomarker measurement, such as for estrogen receptors (ERs), progesterone receptors (PRs), and human epidermal growth factor 2 (HER2), ushered in a new era of breast cancer treatment, with an individual patient’s biomarker profile helping to guide their treatment [3]. More recently, however, multigene molecular tests have provided additional information about prognosis and treatment selection [3], with the Oncotype DX score, a 21-gene RT-PCR assay for ER+ and HER2− patients, leading the way [4,5]. This test provides physicians and patients with a validated recurrence score (RS) that predicts cancer recurrence and adjuvant chemotherapy benefit [6,7,8]. Studies have demonstrated that the Oncotype DX score can more reliably predict prognosis than standard histopathologic features in hormone-positive, HER2-negative patients regardless of axillary nodal status [9,10]. Current guidelines consider patients as high-risk based on two high score thresholds typically used to indicate chemotherapy treatment, which are RS scores greater than either 25 or 30, depending on menopausal status and age [11].

Despite the prognostic benefit conferred by the Oncotype DX score, one study demonstrated that black women in the US were more likely to have a high RS score and to die of axillary-node-negative breast cancer compared to non-Hispanic white women with similar RS scores [12]. The same study also found that the Oncotype DX score has lower prognostic accuracy in black women, suggesting that multigene molecular tests may require calibration in ethnically diverse populations [12].

As an increasing number of multigene molecular tests are developed to guide cancer prognostication and treatment [13], a standardized method for evaluating the performance of the scores in the context of available clinical parameters is necessary. Cox proportional hazard regression has been widely used to evaluate the utility of the Oncotype DX score in breast cancer [14,15]. However, one major limitation of the Cox model is that it fails to capture non-linear relationships, which may provide important prognostic information. For example, variables that change non-linearly with important clinical parameters may not be captured while using the Cox model for survival analysis. In the present study, we investigate the performance of the Cox model in comparison to a random forest survival model [16], which can capture non-linear relationships, to evaluate the ability of the Oncotype DX score to predict chemotherapy benefit on breast-cancer-specific survival (BCSS) across different racial groups. Furthermore, the approach from this study can be generalized to analyze the performance of other multigene molecular tests, as well as to assist with test calibration in racially and ethnically diverse populations.

## 2. Methods

### 2.1. SEER-Oncotype DX Database

We conducted a population-based, retrospective cohort study using the SEER Oncotype DX database, which contains various data for breast cancer patients diagnosed between 2004 and 2015 [17]. The SEER database provides clinical information, including year of diagnosis, race, tumor subtype (luminal A/B or pre-2010), grade (well, moderately, poor, or undifferentiated/anaplastic), stage (I, II, III, IV), ER status, breast-cancer-specific survival, and overall survival. Within the tumor subtype, patients are classified as luminal A, luminal B, or pre-2010, the latter predating the adoption of luminal A/B classification methods. Luminal A patients are hormone receptor (HR)-positive and HER2-negative, while luminal B patients are HR+/HER2+ [18], and the present analysis was restricted to luminal A patients due to their HER2− status. For patients who underwent Oncotype DX testing, the continuous RS score is provided, in addition to risk categories, with the high-risk score greater than 30, the intermediate-risk score between 18–30, and the low-risk score less than 18. In the present study, we focused on ER+, early stage (I or II), luminal A (HR+/HER2−) patients between ages 35 to 80 years at the time of diagnosis (Supplementary Figure S1). Our cohort of lymph-node-negative patients (LN−) with the aforementioned criteria and an available Oncotype DX score included 49,443 patients, with 3178 (6.4%), 16,736 (33.8%), and 29,529 (59.7%) with high-, intermediate-, and low-risk test results, respectively (Supplementary Table S1). Of these patients, 41,685 (84.3%) were white, 4125 (8.3%) were black, and 3633 (7.3%) were Asian. All patients included demonstrated the luminal A subtype and were ER+. Within the cohort, 45,227 (91.5%) of all patients, 38,229 (91.7%) of white patients, 3677 (89.1%) of black patients, and 3321 (91.4%) of Asian patients were PR+. Finally, 8534 (17.3%) of all patients, 7044 (16.8%) of white patients, 818 (19.8%) of black patients, and 672 (18.5%) of Asian patients were treated with chemotherapy.

Our cohort of ER+, early-stage (I or II), luminal A (HR+/HER2−), lymph-node-positive patients (LN+) between ages 35 and 80 years with an available Oncotype DX score included 9858 patients, with 582 (5.9%), 3266 (33.1%), and 6010 (61.0%) with high-, intermediate-, and low-risk test results, respectively (Supplementary Table S1). Of these patients, 8296 (84.2%) were white, 919 (9.3%) were black, and 643 (6.5%) were Asian. All patients included demonstrated the luminal A subtype and were ER+. Within the cohort, 9144 (92.8%) of all patients, 7722 (93.1%) of white patients, 823 (89.6%) of black patients, and 599 (93.2%) of Asian patients were PR+. Finally, 3168 (32.1%) of all patients, 2624 (31.6%) of white patients, 331 (36.0%) of black patients, and 213 (33.1%) of Asian patients were treated with chemotherapy. Given the smaller sample size for the lymph-node-positive cohort, random forest survival modeling analysis was conducted on the larger cohort but not by race.

### 2.2. Survival Analysis

Survival analyses were performed with the R survival (version 3.4.0) and randomForestSRC (version 3.1.1) packages. Univariate and multivariate Cox proportional hazards regression were performed using the “coxph” function, and hazard ratios were extracted from both univariate and multivariate models for Oncotype DX scores. Random forest survival curve models were generated with the “rfsrc” function with 1000 trees; predictions were generated with the “predict.rfsrc” function (Supplementary Figure S1). The random forest survival model is a non-parametric ensemble machine learning (ML) tool that is constructed with multiple independent decision trees (*n* = 1000 in this study), and each tree receives a random subset of samples and then randomly selects a subset of variables at each branch point for prediction purposes [19]. The final prediction made by the model is an average of the prediction of each individual tree [19]. The use of the random forest model bypasses the need to impose constraints (parametric or non-parametric) on the underlying distribution of the data, thereby allowing the random forest survival model to deal with high-level interactions and higher-order terms in variables to improve prediction accuracy [20].

### 2.3. Statistical Analysis

All analyses were performed in R (version 4.2.2). Concordance indices (C-indices) were extracted from univariate and multivariate Cox proportional hazards regression models. Wilcoxon signed-rank tests were calculated using the “compare_means” function from the ggpubr R package (version 0.5.0). A bootstrapping method with 10 iterations using a random selection of 70% of the original data was used to identify the chemotherapy benefit threshold confidence intervals with the precise threshold for each iteration identified using the “optimize” function in R. Average and relative risk plots were generated using loess regression, a non-parametric approach that uses multiple regressions to predict local y-values. Forest plots were generated using the forestploter R package (version 1.0.0).

## 3. Results

### 3.1. Characterizing Important Clinical Parameters with a Linear Cox Proportional Hazards Regression

**Figure 1:**
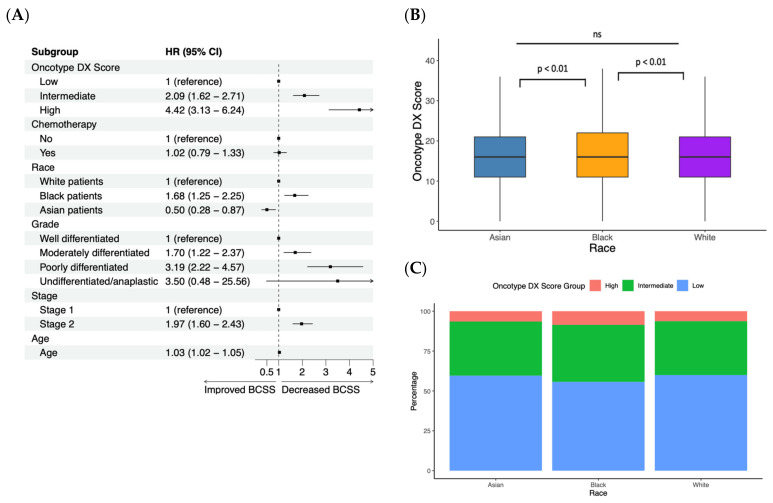
Characterizing important clinical parameters with a linear Cox proportional hazards regression. (**A**) Forest plot demonstrating hazard ratios of breast-cancer-specific survival (BCSS) for various clinical parameters, such as Oncotype DX score, chemotherapy status, race, grade, stage, and age using Cox proportional hazards regression. (**B**) Boxplot demonstrating Oncotype DX score for Asian, black, and white patients with significance determined via Wilcoxon signed-rank tests. Note: “ns” indicates *p* > 0.05. (**C**) Percentage of high, intermediate, and low Oncotype DX score classifications by race. Note: high > 30, intermediate 18–30, and low < 18.

In our cohort of early stage, ER-positive (ER+), HER2-negative (HER2−) lymph-node-negative (LN−) breast cancer patients, a multivariate Cox proportional hazards model was used to investigate the effect of age, stage, grade, tumor subtype, race, Oncotype DX score classification, and chemotherapy status on breast-cancer-specific survival (BCSS). Hazard ratios were extracted from the Cox coefficients and are shown in Figure 1A. High and intermediate Oncotype DX score (vs. low Oncotype DX score), black race (vs. white), poorly/moderately differentiated (vs. well-differentiated), stage II (vs. stage I), and age all exhibited a significantly increased hazard ratio. Asian (vs. white) race demonstrated a significantly decreased hazard ratio, while undifferentiated/anaplastic grade (vs. well-differentiated) and chemotherapy status did not have a significant effect. The incorporation of the Oncotype DX classification improved the model accuracy by assessing the concordance index, a measure of the rank correlation between predicted scores. In a model with the Oncotype DX score, the concordance index (CI) was 0.77 compared to that of the model without it (CI = 0.75) (Supplementary Figure S2A). We then analyzed the Oncotype DX score data in our patient cohort. Black patients had a statistically significant higher Oncotype DX score (*p* < 0.01) compared to white and Asian patients (Figure 1B). Black patients also had a lower percentage of low Oncotype DX classifications and a higher percentage of high Oncotype DX classifications compared to white and Asian patients (Figure 1C). The percentage of black patients with a high Oncotype DX score was 8.6%, compared to 6.3% of white patients and 6.5% of Asian patients. In addition, the Oncotype DX score showed a worse prognostic value in black women (CI = 0.73) than in white (CI = 0.78) and Asian (CI = 0.80) women (Supplementary Figure S2B).

### 3.2. Patients with High Oncotype DX Scores May Benefit from Chemotherapy

**Figure 2:**
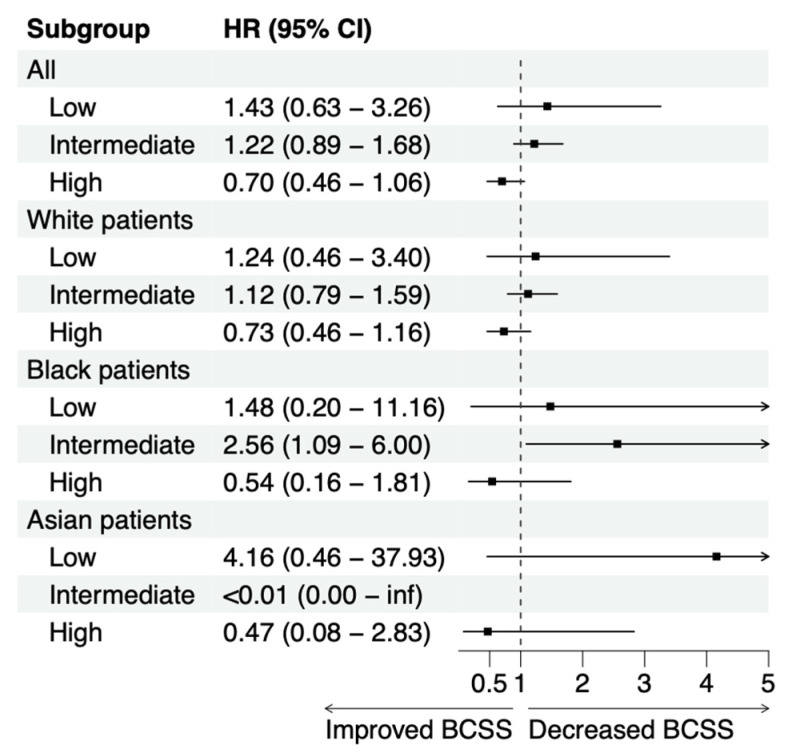
Patients with high Oncotype DX score benefit from chemotherapy Forest plot demonstrating chemotherapy hazard ratio for breast-cancer-specific survival (BCSS) stratified by Oncotype DX score group in all, white, black, and Asian patients.

While the Cox proportional hazards regression is a powerful tool to capture and analyze linear relationships, the model is unable to capture non-linear relationships. As shown in Figure 1A, when all patients were analyzed, receiving chemotherapy was not associated with a survival benefit as the intermediate and high Oncotype DX scores demonstrated hazard ratios of 2.09 (95% CI: 1.62–2.71) and 4.42 (95% CI: 3.13–6.24), respectively. The lack of a benefit is likely due to the fact that patients with unfavorable tumor characteristics are more likely to be treated with chemotherapy. When patients are stratified by the Oncotype DX classification, the 3178 patients in the high Oncotype DX group demonstrate a lower hazard ratio compared to patients in the intermediate and low Oncotype DX groups (Figure 2). While the high Oncotype DX score subgroup hazard ratio does cross 1 in all groups, in the cohort including all patients and the cohort including white patients, a stepwise decrease in the hazard ratio for chemotherapy is seen moving from low to intermediate to high Oncotype DX scores. This stepwise pattern is observed in the all patient and white patient cohorts, but it is not seen in the black or Asian patient cohorts.

### 3.3. Random Forest Survival Model Identifies the Optimal Oncotype DX Score Threshold for Adopting Chemotherapy

**Figure 3:**
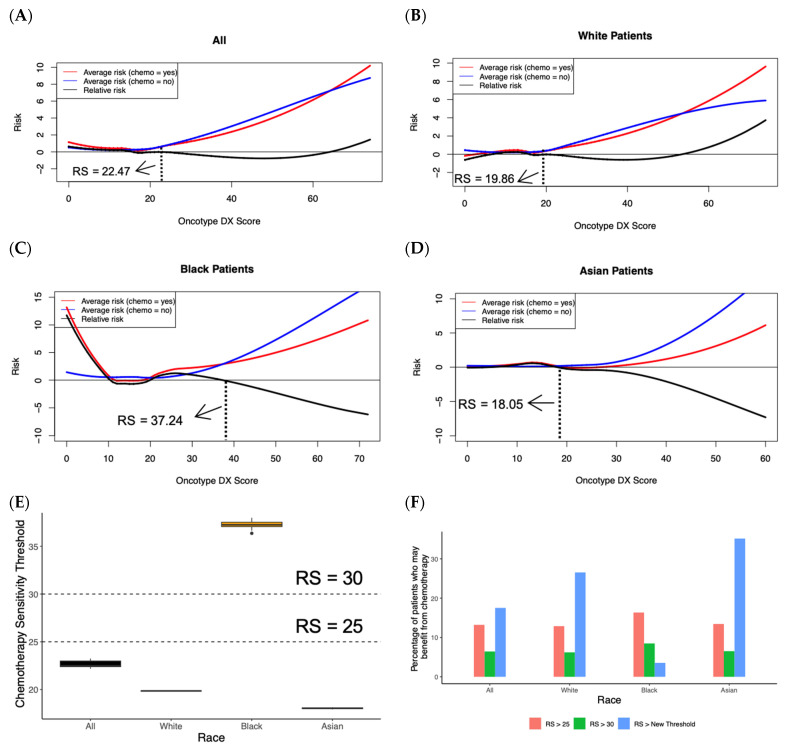
Chemotherapy benefit for all races can be modeled using random forest survival model (**A**–**D**) Average risk and relative risk for all, white, black, and Asian patients using predictions from the random forest survival model. Average risk (chemo = yes) is the random forest survival model’s prediction of BCSS risk upon receiving chemotherapy, while average risk (chemo = no) is the random forest survival model’s prediction of BCSS risk upon not receiving chemotherapy. Dashed black vertical line indicates the chemotherapy benefit threshold Oncotype DX score (RS) for each group. (**E**) Boxplot demonstrating Oncotype DX chemotherapy sensitivity threshold (RS scores) for all, white, black, and Asian patients. All comparisons are significant (*p* < 0.01) via the Wilcoxon signed-rank test. Horizontal lines demonstrate RS > 25 and RS > 30 cutoffs; above these lines, patients may be considered high-risk for recurrence and are recommended to receive chemotherapy. (**F**) Bar graph showing the percentage of patients who might benefit from chemotherapy using the thresholds outlined by the random forest survival model in the present study and the RS > 25 and RS > 30 thresholds traditionally used to define patients at high recurrence risk.

We next built a random forest survival model (see Supplementary Figure S1) using the Oncotype DX score, patient race, age and chemotherapy status to model BCSS and capture non-linear relationships in our cohort of 49,443 stage I/II, ER+, HER2−, LN− breast cancer patients. Based on the fitted model, for each patient, we calculated two risk scores, risk.yes and risk.no, to indicate the predicted BCSS when the patient was treated and not treated by chemotherapy, respectively. Of note, in the real data, a patient can only be either treated or not treated by chemotherapy. However, by leveraging our fitted random forest survival model, we can estimate risk.yes and risk.no by resetting the treatment variable (from yes to no or vice versa) while preserving all the other variables. Plots demonstrating average risk ((risk.no + risk.yes)/2) and relative risk (risk.yes − risk.no) versus Oncotype DX score are shown in Figure 3A–D. The threshold at which patients benefit from chemotherapy occurs when the risk.no line exceeds the risk.yes line in the average risk lines or when the relative risk line crosses zero. Qualitative differences are seen between the cohort, including all patients and cohorts stratified by patient race.

To quantify these differences and identify a confidence interval for the threshold, we performed a bootstrapping analysis by randomly selecting a subset of 70% of the data 10 times and identifying the threshold value for that iteration. For the cohort including all patients, the threshold identifying the point at which the predicted benefit of receiving chemotherapy outweighs the predicted risk of receiving chemotherapy occurs at a median Oncotype DX score of 22.47 (IQR: 22.40–22.99) (Figure 3E). Repeating the analysis separately for white, black, and Asian patients, the thresholds occur at a median Oncotype DX score of 19.86 (IQR: 19.85–19.86), 37.24 (IQR: 37.05–37.52), and 18.05 (IQR: 17.99–18.09), respectively, and all comparisons were significant (*p* < 0.001) using Wilcoxon signed-rank tests. Based on our results, 2.05–2.72-fold more patients from the entire cohort, 2.07–4.27-fold more white patients, 0.42–1.93-fold more black patients, and 2.06–5.39-fold more Asian patients may benefit from receiving chemotherapy compared to the group of patients that the Oncotype DX score currently recommends should receive chemotherapy (RS > 25 and RS > 30, respectively) (Figure 3F).

### 3.4. Random Forest Survival Model Demonstrates Chemotherapy Benefit in LN+ Patients

**Figure 4:**
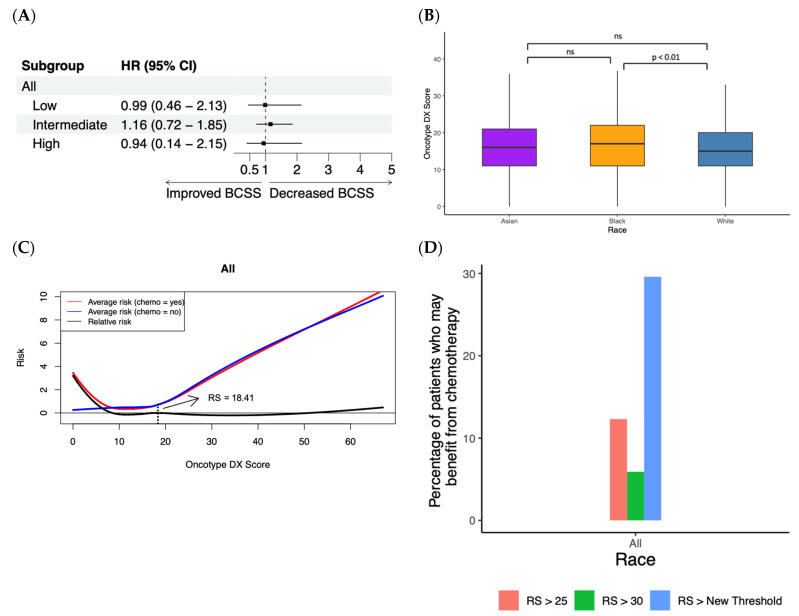
Cox proportional hazards regression versus random forest survival model in LN+ patients. (**A**) Forest plot demonstrating hazard ratios for various clinical parameters using Cox proportional hazards regression. (**B**) Boxplot demonstrating Oncotype DX score for Asian, black, and white patients with significance determined via Wilcoxon signed-rank tests. (**C**) Average risk and relative risk for all patients using predictions from random forest survival model. Dashed black vertical line indicates the chemotherapy benefit threshold Oncotype DX score (RS). (**D**) Bar graph showing the percentage of patients who might benefit from chemotherapy using the thresholds outlined by the random forest survival model in the present study and the RS > 25 and RS > 30 thresholds traditionally used to define patients at high recurrence risk.

In the SEER database, 9858 patients are stage I/II, ER+, HER2−, and LN+ with an Oncotype DX score, and we repeated the above random forest model survival analysis to determine whether the Oncotype DX score can assist with chemotherapy benefit prediction in a LN+ patient cohort. No significant benefit was seen in this patient cohort upon receiving chemotherapy for any of the Oncotype DX groups (Figure 4A) using a Cox proportional hazards regression. Additionally, black patients had a statistically higher Oncotype DX score compared to white patients (*p* < 0.01), but no significant differences were observed between black and Asian patients or white and Asian patients (Figure 4B). We then built a random forest survival model using the Oncotype DX score, patient race, age and chemotherapy status to model BCSS and capture non-linear relationships in this cohort. Bootstrapping analysis was performed as described above with the chemotherapy benefit threshold occurring at a median Oncotype DX score of 18.41 (IQR: 17.27–19.88) for all patients in this cohort, and the average and relative risk plots are shown in Figure 4C. Based on these results, 2.08-fold more patients from this cohort may benefit from receiving chemotherapy compared to the group of patients with an RS > 25 (Figure 4D). Additionally, when using the RS > 30 cutoff utilized to define high-risk patients in the SEER database, 5.02-fold more patients from this cohort may benefit from receiving chemotherapy.

## 4. Discussion

Multigene molecular tests have emerged in breast cancer treatment as promising tools for patient prognosis and treatment selection. Specifically, the Oncotype DX score is a widely used gene expression test that helps physicians tailor treatment to the individual patient, and after controlling for tumor stage and lymph node status, Oncotype DX users demonstrate statistically longer survival times compared to nonusers [4]. Breast cancer chemotherapy is highly morbid, with long-term side effects including insomnia, cardiotoxicity, fertility and sexual health problems, fatigue, and peripheral neuropathy, among others [21]. From 2004 to 2015, as the use of the Oncotype DX score in ER+ patients increased from 1.5% to 34%, chemotherapy usage decreased from 42% to 36% [4]. The decrease in usage is primarily driven by decreased chemotherapy in patients with a low RS score [4].

However, differences in prognostic benefit of the Oncotype DX score across racial groups have been demonstrated [12,22,23], suggesting tests may require calibration in racially and/or ethnically diverse populations. A separate study found that in women with LN− tumors, higher breast-cancer-specific mortality was seen for black women compared to non-Hispanic white women after accounting for recurrence score risk stratum [12]. Furthermore, the study found that the prognostic accuracy of the recurrence score was significantly lower for black women [12]. Presently, we demonstrate that a random forest survival model that includes the Oncotype DX score, patient race, age, and chemotherapy status can capture important non-linear relationships that a Cox proportional hazards regression may fail to capture. Specifically, we demonstrate a reproducible chemotherapy benefit threshold, at which the predicted benefit of chemotherapy outweighs the predicted risk for all patients in our cohort, in addition to subcohorts for white, black, and Asian patients. In stage I/II, ER+, LN− patients, this threshold occurred at an Oncotype DX score of ~22.5 for all patients, ~20 for white patients, ~37 for black patients, and ~18 for Asian patients.

Notably, the chemotherapy benefit threshold from this study for the cohort including all patients occurs beneath the two high score thresholds typically used to indicate chemotherapy treatment, which are above 30 or 26 or higher [11]. Taking the threshold used by the SEER database for identifying patients at a high risk for relapse (RS > 30), our results suggest that 2.72-fold more patients from the entire LN− cohort, 4.27-fold more white patients, 0.42-fold fewer black patients, and 5.39-fold more Asian patients may benefit from receiving chemotherapy compared to the group of patients that the Oncotype DX score currently recommends should receive chemotherapy. Taken together, these results suggest that specific groups of patients may benefit from chemotherapy at lower Oncotype DX scores while other groups of patients may be exposed to additional risk by receiving chemotherapy at lower Oncotype DX scores. Furthermore, compared to the arbitrarily set points that may define different risk groups, our study provides a quantitative approach to identifying the Oncotype DX score at which patients do or do not benefit from receiving chemotherapy. This is a repeatable method that can be applied to the Oncotype DX score and more broadly to other multigene molecular tests.

Interestingly, according to our models, black women definitively benefit from chemotherapy only at higher Oncotype DX scores, compared to white and Asian women. This prompts a follow-up question: Why do different racial groups have different thresholds for the use of the Oncotype DX tool for chemotherapy selection? For example, why does the Oncotype DX score have lower prognostic value as indicated by the c-index in both previous studies [12] and our present study, in addition to having a higher Oncotype DX score threshold? One possible explanation is that the gene set used to build the algorithm for determining the Oncotype DX score using tumors from participants enrolled in the National Surgical Adjuvant Breast and Bowel Project B-20 trial [24,25] and from single-institution case series [26] may not have been representative of the target population. Using representative cohorts for discovery and validation steps in biomarker development is an important step for ensuring prognostic accuracy and generalizability across diverse populations [27], and while the specific racial/ethnic distribution of patients used to build the Oncotype DX score was not reported, only 6% of B-20 participants were black [24]. An additional explanation that may explain the racial variation in chemotherapy benefit using the Oncotype DX score is that the 21-gene expression panel may fail to account for various host factors that play an important role in cancer outcomes, such as BMI [28], smoking [29], or screening mammography utilization [30].

While the Oncotype DX score is typically used for LN− patients, there were 9858 patients in the SEER database with stage I/II, ER+, LN+ disease who received an Oncotype DX score. One study compared RS results among patients with LN−, micrometastatic, and macrometastatic disease, finding a similar RS distribution among the three groups [9]. The RxPONDER trial demonstrated that premenopausal women with one to three positive lymph nodes and an RS score of 25 or lower who received chemoendocrine therapy had longer invasive disease-free survival than those who received endocrine-only therapy, whereas postmenopausal women did not benefit from adjuvant chemotherapy [31]. In our study, LN+ patients include both those with micrometatstatic and macrometastatic disease, and our random forest survival model predicted a benefit from chemotherapy in these patients. In stage I/II, ER+, LN+ patients, the threshold occurred at ~18.5 for all patients, though a smaller sample size for LN+ patients prevented race-specific analysis. Based on these results, 2.08–5.02-fold more patients from this cohort may benefit from receiving chemotherapy compared to the group of patients that the Oncotype DX score currently recommends should receive chemotherapy (RS > 25 or RS > 30, respectively). Interestingly, the magnitude of the difference between the risk.yes and risk.no curves for lymph-node-positive patients was lower than the same difference for lymph-node-negative patients, suggesting that lymph-node-positive patients may benefit from receiving chemotherapy, though the magnitude of the benefit may be less than their lymph-node-negative counterparts. Together, these results suggest that there is some benefit to receiving chemotherapy in select patients with a low RS score, though further analysis should include menopausal status to assist with prediction.

Although our study provides insight into optimizing the clinical use of the Oncotype DX score, one limitation is that chemotherapy usage tends to be underreported in the SEER cohort given that some patient data states “no/unknown” for chemotherapy status, suggesting there are patients who received chemotherapy but were listed as “unknown.” Furthermore, the majority of analyzed patients were white, and the sample sizes for black and Asian populations were lower. Future studies will benefit from utilizing data containing additional underrepresented populations. Additionally, the current study is not able to assess for confounders, such as BMI, menopausal status, or access to screening healthcare, among other potential confounding variables. Finally, European Society for Medical Oncology (ESMO) guidelines recommend a method to stratify luminal B patients as HER2+ or HER2− [32]. In the present study, only luminal A/HER2− negative patients were studied due to the structure of the available data, but future studies may repeat this analysis for HER2− patients across both luminal A and luminal B subtypes.

The present study provides a method to identify chemotherapy benefit thresholds with multigene molecular tests using a random forest survival model. Future studies may apply this method to other tests to identify racial or other parameters that may affect benefit thresholds. Additionally, future studies may include radiotherapy as a variable of interest to better understand the interplay of the Oncotype DX score and radiotherapy in breast cancer. Finally, studies may leverage neoadjuvant patient data with clinical response information, multigene molecular test data, and the random forest survival model to predict patient response, thereby eliminating the need for creating datasets for predicting the risk of receiving or not receiving chemotherapy in future studies.

## 5. Conclusions

The models from our present study suggest that physicians may be able to provide chemotherapy at lower Oncotype DX scores than current guidelines suggest. By leveraging a random forest survival model and twelve years of survival data from the SEER database, our study shows that over 2-fold more lymph-node-negative (LN−) and 2–5-fold more lymph-node-positive (LN+) patients who are not recommended for chemotherapy by their Oncotype DX test result may have the potential to benefit from chemotherapy. More broadly, we have provided a reproducible method to identify and calibrate chemotherapy benefit thresholds for multigene molecular tests in cancer.

## Data Availability

Publicly available datasets were analyzed in this study. This data can be found here: https://seer.cancer.gov/ (accessed on 1 January 2021).

## Supplementary Materials

The following supporting information can be downloaded at: https://www.mdpi.com/article/10.3390/cancers15123217/s1, Figure S1: Random forest survival model; Figure S2: Concordance indices by Oncotype DX score and race; Table S1: Demographic data.
