# Supplementary material for: Predicting Chemotherapy Benefit across Different Races in Early-Stage Breast Cancer Patients Using the Oncotype DX Score
Source: Cancers (Basel). 2023 Jun 16;15(12):3217. doi: 10.3390/cancers15123217 (PMC10296905; doi:10.3390/cancers15123217)
Supplement: Supplementary file 1 [file cancers-15-03217-s001.zip › cancers-2404570-supplementary.pdf]

TITLE: Predicting Chemotherapy Benefit across Different Races in Early-Stage Breast Cancer Patients Using the Oncotype DX Score (Supplementary materials)

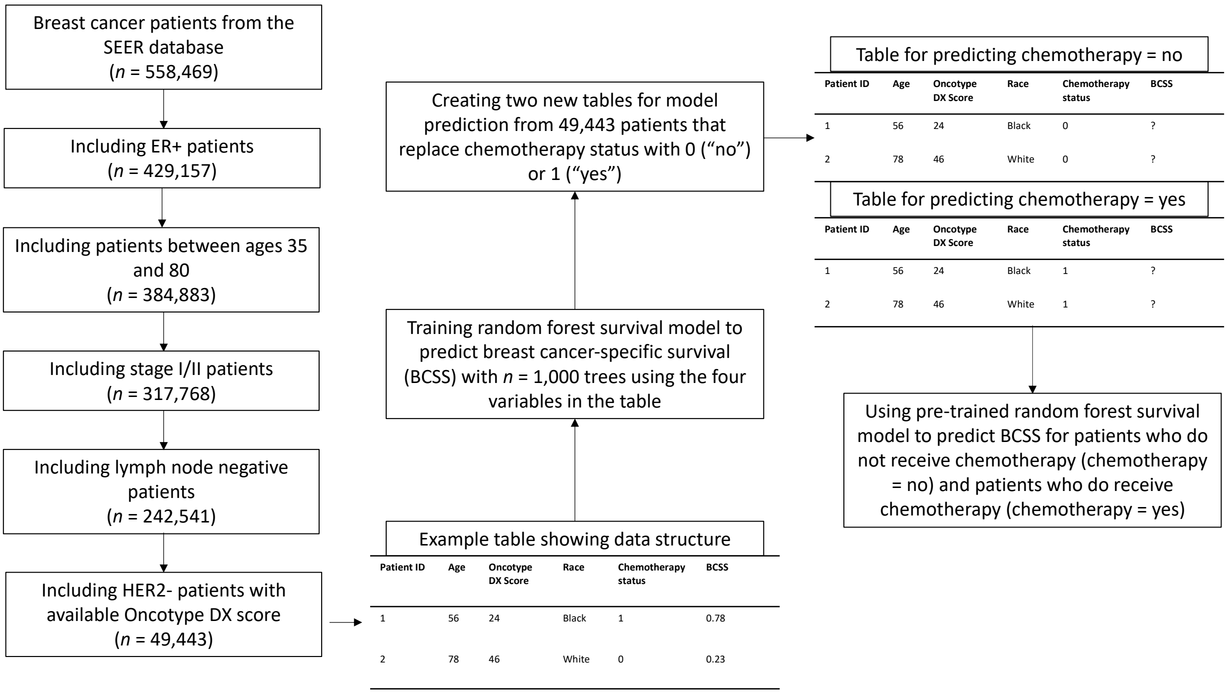

Supplementary Figure S1. Random forest survival model. Diagram demonstrating the set-up of the random forest survival model for the lymph node negative cohort of patients.

Supplementary Table S1. Demographic data Demographic data for stage I/II, ER+, LN-/LN+ patient cohorts, including Oncotype DX group, Oncotype DX score, tumor subtype, patient age, tumor grade and stage, and chemotherapy status.

Patient cohorts	Race	OncoDX group	OncoDX score (median, IQR)	Subtype	Age (median, IQR)	Grade (differentiation status)	Stage	ER/PR status	Chemotherapy status
Stage I/II, ER+, LN-	All (n = 49,443)	High (n = 3,178)<br>Intermediate (n = 16,736)<br>Low (n = 29,529)	16 (11 – 21)	Luminal A (n = 49,443)	59 (50 – 66)	Well (n = 14,637)<br>Moderately (n = 26,326)<br>Poorly (n = 7,472)<br>Undifferentiated/anaplastic (n = 36)	I (n = 37,471)<br>II (n = 11,972)	ER+ (n = 49,443)<br>PR+ (n = 45,227)	Yes (n = 8,534)
	White (n = 41,685)	High (n = 2,591)<br>Intermediate (n = 14,032)<br>Low (n = 25,062)	16 (11 – 21)	Luminal A (n = 41,685)	59 (51 – 66)	Well (n = 12,598)<br>Moderately (n = 22,116)<br>Poorly (n = 6,117)<br>Undifferentiated/anaplastic (n = 31)	I (n = 31,899)<br>II (n = 9,786)	ER+ (n = 41,685)<br>PR+ (n = 38,229)	Yes (n = 7,044)
	Black (n = 4,125)	High (n = 350)<br>Intermediate (n = 1,473)<br>Low (n = 2,302)	16 (11 – 22)	Luminal A (n = 4,125)	58 (50 – 65)	Well (n = 1,070)<br>Moderately (n = 2,193)<br>Poorly (n = 764)<br>Undifferentiated/anaplastic (n = 1)	I (n = 3,022)<br>II (n = 1,103)	ER+ (n = 4,125)<br>PR+ (n = 3,677)	Yes (n = 818)
	Asian (n = 3,633)	High (n = 237)<br>Intermediate (n = 1,231)<br>Low (n = 2,165)	16 (11 – 21)	Luminal A (n = 3,633)	55 (48 – 63)	Well (n = 969)<br>Moderately (n = 2,017)<br>Poorly (n = 591)<br>Undifferentiated/anaplastic (n = 4)	I (n = 2,550)<br>II (n = 1,083)	ER+ (n = 3,633)<br>PR+ (3,321)	Yes (n = 672)
Stage I/II, ER+, LN+	All (n = 9,858)	High (n = 582)<br>Intermediate (n = 3,266)<br>Low (n = 6,010)	16 (11 – 21)	Luminal A (n = 9,858)	59 (51 – 67)	Well (n = 2,571)<br>Moderately (n = 5,613)<br>Poorly (n = 1,504)<br>Undifferentiated/anaplastic (n = 9)	I (n = 388)<br>II (n = 9,470)	ER+ (n = 9,858)<br>PR+ (n = 9,144)	Yes (n = 3,168)
	White (n = 8,296)	High (n = 444)<br>Intermediate (n = 2,719)<br>Low (n = 5,133)	15 (11 – 20)	Luminal A (n = 8,296)	60 (51 – 67)	Well (n = 2,238)<br>Moderately (n = 4,719)<br>Poorly (n = 1,202)<br>Undifferentiated/anaplastic (n = 8)	I (n = 325)<br>II (n = 7,971)	ER+ (n = 8,296)<br>PR+ (n = 7,722)	Yes (n = 2,624)
	Black (n = 919)	High (n = 84)<br>Intermediate (n = 335)<br>Low (n = 500)	17 (11 – 23)	Luminal A (n = 919)	60 (51 – 67)	Well (n = 214)<br>Moderately (n = 493)<br>Poorly (n = 183)<br>Undifferentiated/anaplastic (n = 0)	I (n = 34)<br>II (n = 885)	ER+ (n = 919)<br>PR+ (n = 823)	Yes (n =331)
	Asian (n = 643)	High (n = 54)<br>Intermediate (n = 212)<br>Low (n = 377)	16 (11 – 21)	Luminal A (n = 643)	55 (47 – 64)	Well (n = 119)<br>Moderately (n = 401)<br>Poorly (n = 119)<br>Undifferentiated/anaplastic (n = 1)	I (n = 29)<br>II (n = 614)	ER+ (n = 643)<br>PR+ (n = 599)	Yes (n = 213)

(**A**) (**B**)

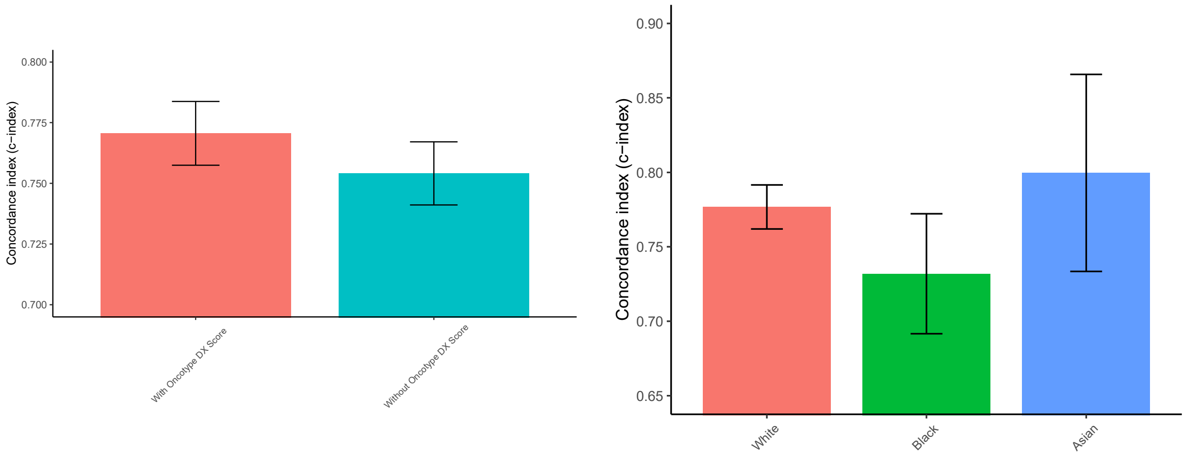

Supplementary Figure S2. Concordance indices by Oncotype DX score and race (**A**) Plot showing concordance index (+/-) standard error for a Cox proportional hazards regression including and excluding the Oncotype DX score (**B**) Plot showing concordance index (+/-) standard error for a Cox proportional hazards regression including the Oncotype DX score for datasets stratified by white, black, and Asian patients.
